# Users’ experiences of wearable activity trackers: a cross-sectional study

**DOI:** 10.1186/s12889-017-4888-1

**Published:** 2017-11-15

**Authors:** Carol Maher, Jillian Ryan, Christina Ambrosi, Sarah Edney

**Affiliations:** Alliance for Research in Exercise, Nutrition and Activity (ARENA), School of Health Sciences & Sansom Institute for Health Research, University of South Australia, GPO Box 2471, Adelaide, South Australia 5001 Australia

**Keywords:** Fitness, Health behaviour, Measurement, Motion sensors, Pedometry, Physical activity assessment

## Abstract

**Background:**

Wearable activity trackers offer considerable promise for helping users to adopt healthier lifestyles. This study aimed to explore users’ experience of activity trackers, including usage patterns, sharing of data to social media, perceived behaviour change (physical activity, diet and sleep), and technical issues/barriers to use.

**Methods:**

A cross-sectional online survey was developed and administered to Australian adults who were current or former activity tracker users. Results were analysed descriptively, with differences between current and former users and wearable brands explored using independent samples *t*-tests, Mann-Whitney, and chi square tests.

**Results:**

Participants included 200 current and 37 former activity tracker users (total *N* = 237) with a mean age of 33.1 years (SD 12.4, range 18–74 years). Fitbit (67.5%) and Garmin devices (16.5%) were most commonly reported. Participants typically used their trackers for sustained periods (5–7 months) and most intended to continue usage. Participants reported they had improved their physical activity (51–81%) more commonly than they had their diet (14–40%) or sleep (11–24%), and slightly more participants reported to value the real time feedback (89%) compared to the long-term monitoring (78%). Most users (70%) reported they had experienced functionality issues with their devices, most commonly related to battery life and technical difficulties.

**Conclusions:**

Results suggest users find activity trackers appealing and useful tools for increasing perceived physical activity levels over a sustained period.

**Electronic supplementary material:**

The online version of this article (10.1186/s12889-017-4888-1) contains supplementary material, which is available to authorized users.

## Background

Physical inactivity and poor diet are leading modifiable causes of death and disease globally. They increase the risk of developing chronic health conditions -- such as cardiovascular disease, type 2 diabetes, obesity, cancers, depression and anxiety -- leading to premature death and reduced quality of life [1, 2]. In addition, poor sleep quality and insufficient sleep quantity have been linked to neuroendocrine dysfunction, increasing the risk of obesity [3], stress, cardiovascular disease and mood disorders [4]. Further efforts are needed to assist people to adopt healthier lifestyles.

Wearable activity trackers offer considerable promise for assisting individuals to improve lifestyle behaviours such as physical activity, diet and sleep. In the past 5 years, a wide variety of manufacturers and models have entered the consumer activity tracker market, such as Fitbit, Garmin and Jawbone. Typically, the trackers are worn on the wrist, and come with a smartphone app (and sometimes also online software) to allow users to track their activities relating to physical activity, sleep and sometimes diet, over a prolonged period. From 2010 to 2015, Fitbit sold over 38 million devices worldwide [5], and in 2017, it is estimated that healthcare wearable device sales will total 870 million US dollars [6].

Early research concerning consumer activity trackers has focussed on device accuracy and validity. Taken together, this research suggests that activity trackers are reasonably accurate, though validity varies for different manufacturers and models [7], and according to the health behaviour metric in question. To date, evidence suggests that activity trackers are most accurate for measuring steps, and less so for other physical activity-related metrics (e.g. stairs climbed, energy expenditure) and sleep [8]. Fitbit devices have most commonly been researched and typically perform strongly.

However, such studies have generally been carried out over a short period (e.g. 1–7 days) [8], and very little research has scrutinised the utility of activity trackers in the longer term. In addition, such studies have typically not required participants to use the manufacturers’ apps or software, therefore, little is known about the usability of such software. Furthermore, practical issues such as durability, longevity and comfort have not been scrutinised, though preliminary evidence suggests they can be problematic with particular models. For example, Ferguson reported issues with the Nike Fuelband battery not holding sufficient charge and the Jawbone Up device failure after 4 weeks of use [9].

Understanding the users’ experience is vital for clinicians asked to provide advice regarding activity trackers, and researchers and service providers seeking to use activity trackers with clinical or population groups. This study aimed to address this gap in the literature by exploring users’ experience of activity trackers, including the perceived usefulness of devices for tracking and modifying lifestyle behaviours (physical activity, diet and sleep), ease of use, patterns of usages, and barriers to use.

## Methods

Ethical approval for this cross-sectional, descriptive online survey was provided by the University of South Australia Human Research Ethics Committee. Data collection took place in April to May 2016. As per ethical requirements, the cover page of the survey contained detailed information about the study, and completion of the online survey was regarded as informed consent. A complete copy of the survey instrument is provided in Additional file 1: Supplementary Material 4.

### Participants

Participants were required to be aged over 18 years, living in Australia, and either currently using, or having formerly used, an activity tracker. This study aimed to capture users of smart activity trackers, and a wide range of activity trackers, fitness devices and related technologies are now available. As such, the following exclusion criteria were applied: use of an activity tracker smartphone app without an associated wearable activity tracker, use of a fitness watch which could not measure daily steps, and activity trackers that cannot interact with a computer or smart phone.

### Instrument development

A purpose-designed survey instrument was developed to address the research objectives. Three independent experts in the field provided minor feedback regarding the survey’s structure, content and wording. Test-retest reliability was evaluated in a sample of *n* = 11 across a time interval ranging from seven and 21 days (mean = 10, SD = 4.6). Bivariate correlation coefficients between test scores ranged between *r* = 0.30 to 0.81 for survey sections. Items that were measured on a 5-point Likert scale all included response options of 1 = “Disagree strongly”, 2 = “Somewhat disagree”, 3 = “Neutral”, 4 = “Somewhat agree”, 5 = “Agree strongly”.

### Demographic characteristics and activity tracker usage

All participants were asked for basic demographic characteristics, including sex, age, education level (high school, TAFE/technical college, undergraduate degree, or postgraduate degree) and relationship status (single, in a relationship, or unspecified). Participants were also asked whether they were currently using an activity tracker or had formerly used an activity tracker, and which brand of activity tracker they currently or formerly used (open-ended). In addition, participants were asked how long they had worn their activity trackers, and if they were current users of an activity tracker, how long they intended to continue wearing it into the future.

### Data usage and sharing

Three items were included to assess how participants used and shared the data derived from their activity trackers. In two items, participants were asked to indicate the extent to which they found the long term and short term monitoring on the activity tracker useful, with responses recorded on a 5-point Likert scale. Participants who were current users were also asked whether they used social features of their trackers, and if so, on which social media platform. Participants could select from six options, including “Facebook”, “Twitter”, “Instagram”, “within the activity tracker’s software”, “I don’t share my activity data”, or “other (please specify)”.

### Behaviour change

Four items were included to assess perceived behaviour change related to use of the activity tracker. Three items asked participants to indicate the extent to which they agree that the activity tracker had helped them to eat healthier, increase physical activity, and sleep more, with responses recorded on a 5-point Likert scale. The fourth item offered participants a selection of graphs, and asked them to indicate which one most accurately reflected the change in their physical activity level since using an activity tracker.

### Motivation for wearing an activity tracker

Participants were asked to identify whether wearing their activity tracker motivated them in eight different domains, including: ‘improve fitness’, ‘improve health’, ‘improve appearance’, ‘lose weight’, ‘monitor activities’, ‘share activities’, ‘compete with family or friends’, and ‘keep up with technology’, with responses recorded on a 5-point Likert scale.

### Practical issues

Up to three questions explored practical issues related to use of activity trackers. In one item, participants were asked to select any complaints about their activity trackers, including responses such as ‘technical issues’, ‘low battery life’, ‘it often does not match my outfit’, with an open-ended ‘other’ option included to capture additional complaints. In the second item, participants were asked to indicate the extent to which they agreed their activity tracker experience had been positive, with responses recorded on a 5-point Likert scale. Finally, participants who had formerly worn an activity tracker were asked to select a reason why they had ceased to use it, from 10 options including reasons such as ‘it broke’, it was difficult to understand’, or ‘it wasn’t helping with my goals’, with an open-ended ‘other’ option included to capture additional reasons.

### Perceptions of features

Twenty-one items were included to assess current users’ perceptions of the ease of use, usefulness, and accuracy of seven common features of activity trackers: active minutes, step counts, stair counts, sleep, heart rate, energy burned, and energy consumed, with responses recorded on a 5-point Likert scale.

### Procedure

The online survey was delivered via Survey Monkey. Recruitment was promoted using low-cost distribution methods on Facebook and Twitter, primarily; sharing the survey link with a variety of Facebook community groups in the field of health and fitness (e.g. sporting clubs, cycling interest groups). Additionally, the survey link was shared on the University of South Australia’s Facebook and Twitter feeds, and shared by individual members of the research team. An incentive (in the form of a $50 voucher random prize draw) was offered to encourage people to share the social media posts. In addition, a second $50 voucher random prize draw was offered for people who completed the survey (there was an optional item at the end of the survey requesting their email address if they wished to be entered into the prize draw).

### Analysis

Data were downloaded into SPSS Version 21. Categorical variables were analysed using frequency of responses and percentages, and continuous variables were analysed using medians, means, ranges, and standard deviations. Differences between former and current users were explored using independent samples *t*-tests (continuous, parametric data), Mann-Whitney U tests (continuous, non-parametric data), and chi square tests (categorical data). Likewise, differences in use and experience on the basis of activity tracker manufacturer were examined. For these analyses, there were only sufficient numbers of responses to facilitate comparison of Fitbit versus Garmin devices, with small numbers of users reported for other device manufacturers.

## Results

### Demographic characteristics and activity tracker usage

Three hundred and five people began the survey; however, 67 individuals did not complete it, and one individual was aged <18 years, resulting in a final sample of 237 participants. Of these, 200 participants were currently using an activity tracker and 37 participants were former users. Participants’ characteristics are presented in Table 1.

**Table 1:** Demographic characteristics of participants

Demographic Characteristic	Former user (*n* = 37)	Current users (*n* = 200)	Total (*N* = 237)	*p*
	*n* (%)	*n* (%)	*n* (%)	
Sex				.76
Male	10 (27.0%)	59 (29.5%)	69 (29.1%)	
Female	27 (73.0%)	141 (70.5%)	168 (70.9%)	
Age^a^				.10
18–24	18 (51.4%)	57 (30.5%)	75 (33.8%)	
25–34	5 (14.3%)	51 (27.2%)	56 (25.2%)	
35–44	6 (17.2%)	41 (21.9%)	47 (21.2%)	
45–54	5 (14.3%)	23 (12.3%)	28 (12.6%)	
55–64	1 (2.9%)	13 (6.9%)	14 (6.3%)	
65>	–	2 (1.1%)	2 (1.0%)	
Education				.22
High School	12 (32.4%)	40 (20.0%)	52 (21.9%)	
TAFE/certificate/diploma/apprenticeship	4 (10.8%)	38 (19.0%)	42 (17.7%)	
Undergraduate degree	12 (32.4%)	83 (41.5%)	95 (40.1%)	
Postgraduate degree	9 (24.3%)	39 (19.5%)	48 (20.3%)	
Relationship Status				.01
In a relationship	17 (45.9%)	137 (68.5%)	154 (65.0%)	
Single	16 (43.2%)	48 (24.0%)	64 (27.0%)	
Unspecified	4 (10.8%)	15 (7.5%)	19 (8.0%)	

Note: ^a^ *n* = 222 due to missing data

On average, participants were aged 33.1 years (SD 12.4, range 18–70 years), and approximately two thirds were female. There were no sociodemographic differences between current and former users with the exception that current users were more likely to be in a relationship than former users (*X* ^2^ (2) = 8.98, *p* = .01).

The most commonly used brand of activity tracker was Fitbit (67.5%, *n* = 160), followed by Garmin (16.5%, *n* = 39), Apple (3.4%, *n* = 8), Jawbone (2.5%, *n* = 6), Samsung (1.7%, *n* = 4), Polar (1.3%, n = 3), and other (7.1%, *n* = 17). Overall, users were most likely to obtain their activity trackers by purchasing it themselves (56.58%) or receiving it as a gift from their family (43.5%).

Participants were asked how long they had been using their activity trackers. Former users wore their activity trackers for a median duration of 5 months (*range* 0–24), whilst current users reported wearing their activity trackers for a median duration of 7 months (*range* 0 - >36 months). When current users were asked how long they planned to use their activity tracker into the future, the median response was equal to the maximum allowed response to this question, or >36 months (range = 0 to >36 months).

### Motivation and behaviour change

Overall, activity trackers mainly motivated users to monitor activity patterns (35.9%, *n* = 85), improve fitness (27.4%, *n* = 65) and improve health (18.1%, *n* = 43) (see Additional file 2: Table S1 for full details). Participants were asked a series of questions about the usefulness of the activity data collected by their tracker. The majority of current users either strongly or somewhat agreed that various features on their trackers were useful, including: steps (95%), active minutes (76%), sleep (66%), heart rate (63%), stairs climbed (58%), energy burned (57%). Fewer agreed that the food intake feature was useful (36%). Participants were also asked their opinions on the usefulness of real time monitoring and long term monitoring features. The majority of current (89%) and former (54%) users agreed that the real time monitoring was useful, while slightly fewer current users (78%) and former users (41%) agreed that long term monitoring was useful.

Participants were asked their perceptions on whether they had changed their activity patterns as a result of wearing their activity tracker. The majority of current (81.4%) and former (51.3%) users believed that they incorporated more physical activity into their day whilst wearing their activity tracker. In contrast, only 40.2% of current users and 13.5% of former users reported they had improved their eating patterns as a results of using their tracker, and even fewer reported they had changed their sleeping pattern (24.1% of current users and 10.8% of former users). In regards to physical activity specifically, participants were asked to identify which behavioural pattern most accurately represented their physical activity change since they began wearing an activity tracker. Overall, 68.3% of current users and 70.2% of former users reported an initial increase in their activity levels. However, 9.5% of current users and 27.0% of former users reported that this increase was transient, and that their activity levels had subsequently declined to baseline levels.

### Social features and data sharing

Use of social features and data sharing to general social networks (Facebook, Instagram, and Twitter) and to special interest social networks (e.g. Strava – a social network focussed on athletic activities, or the devices’ home platform) was explored amongst current tracker users (Table 2). The majority of participants reported that they did not use social features (65%) nor did they share their activity data on social media platforms (77%). Amongst those who did report using social features, the most common platform for this was the trackers’ support software (35%), and relatively few users reported sharing their data to external social networking sites such as Strava, Facebook or Instagram (1–5%). The prime motivation for using social features was reportedly “to compete with friends” (17%).

**Table 2:** Sharing platforms and reasons for sharing activity tracker data

Sharing platform	Current users, *n* = 200 *n* (%)	
Do not share data	130	(65.0%)
Tracker support software	70	(35.0%)
Strava	9	(4.5%)
Facebook	7	(3.5%)
Instagram	2	(1.0%)
In person	2	(1.0%)
Twitter	1	(0.5%)
Other	3	(1.5%)
Reasons for sharing on social media		
Do not share data on social media	154	(77.0%)
To compete with friends	33	(16.5%)
To share progress	18	(9.0%)
To motivate others	17	(8.5%)
To get encouragement from others	13	(6.5%)
Other	4	(2.0%)

Note: Participants were allowed to select multiple responses, and percentages reflect the number of participants who selected each response option as a portion of all participants in that subgroup

A chi square test was conducted to determine whether participants who shared their activity tracker data via social media reported positive behaviour change more frequently than participants who did not share their information. The results suggested that sharing data via social media was not associated with behaviour change, *X* ^2^ (1) = 1.07, *p* = .30.

### Technical issues and barriers

Overall, 94% of current users and 65% of former users agreed that they had had a positive overall experience using their activity tracker. A Mann-Whitney U test revealed this differed significantly, with current users (*Mdn* = 5.00) more likely to report a positive experience than former users (*Mdn* = 4.00), *U* = 1746.50, *z* = −5.79, *p* = < .001, *r* = .38. Despite this, most users reported technical issues or other complaints relating to their activity trackers (Table 3), most commonly relating to the tracker not suiting their outfit (19%), low battery life (19%), difficulties with the support software (17%) or perceived inaccuracy of data collected (17%). Former users reported more issues than current users overall (*U* = 1648.5, *z* = − 2.36, *p* = .02, *r =* .18), with complaints typically relating to battery life (30%), difficulties with the support software (30%), the device falling off (22%) and wear and tear issues (22%).

**Table 3:** Participants’ complaints regarding activity trackers

Complaints	Former (*n* = 37)		Current (*n* = 200)			Total (*N* = 237)	Significance of between group differences
	*n (%)*		*n (%)*			*N (%)*	*p*
None	6	(16.2%)	65	(32.5%)	71	(30.0%)	.047*
Low battery life	11	(29.7%)	37	(18.5%)	48	(20.0%)	.12
Problems uploading data to supporting software	11	(29.7%)	34	(17.0%)	45	(19.0%)	.07
Does not go with my outfit	6	(16.2%)	38	(19.0%)	44	(18.6%)	.69
Inaccurate at recording data	6	(16.2%)	33	(16.5%)	39	(16.5%)	.97
General wear and tear	8	(21.6%)	24	(12.0%)	32	(13.5%)	.12
Technical issues	6	(16.2%)	23	(11.5%)	29	(12.2%)	.42
Uncomfortable	6	(16.2%)	14	(7.0%)	20	(8.4%)	.10
Falls off	8	(21.6%)	9	(4.5%)	17	(7.2%)	<.001*
Problem navigating supporting website/technology	2	(5.4%)	14	(7.0%)	16	(6.8%)	1.00
Other	2	(5.4%)	12	(6.0%)	14	(5.9%)	1.00
Problems with screen	3	(8.1%)	7	(3.5%)	10	(4.2%)	.19
Problem interpreting the data	3	(8.1%)	7	(3.5%)	10	(4.2%)	.19
Skin irritation	1	(2.7%)	5	(2.5%)	6	(2.5%)	1.00
Lost it	3	(8.1%)	2	(1.0%)	5	(2.1%)	.03*
Waterproof		–	4	(2.0%)	4	(1.7%)	1.00
Cleaning	1	(2.7%)	2	(1.0%)	3	(1.3%)	.40

Note. ^*^ = *p <* .05; Participants were allowed to select multiple responses, and percentages reflect the number of participants who selected each response option as a portion of all participants in that subgroup

Former users were asked to identify why they no longer use their activity tracker, and the main reasons given were that they felt they had learnt everything they could from their tracker (30%), their tracker was broken (22%), and/or their tracker was not helping them achieve their goals (14%; Table 4).

**Table 4:** Former users’ reasons for no longer wearing their activity tracker

Reasons to stop wearing tracker	Former users (*n* = 37) *n* (%)	
“I learnt everything I could”	11	(29.7%)
Broken tracker	8	(21.6%)
It was not helping achieve goals	5	(13.5%)
Technical difficulties	4	(10.8%)
Got lost	4	(10.8%)
Forgot to charge it	4	(10.8%)
Experiencing negative psychological impact	3	(8.1%)
Intrusive	3	(8.1%)
Difficult to understand	2	(5.4%)
Did not like it	2	(5.4%)
Inconvenient	2	(5.4%)
Forgot to put it back on after taking it off	2	(5.4%)
Information was not important	2	(5.4%)
Work	2	(5.4%)
Annoying to charge	1	(2.7%)
Became too dependent	1	(2.7%)
Band continuously came undone	1	(2.7%)
Limited functionality	1	(2.7%)

Note: Participants were allowed to select multiple responses, and percentages reflect the number of participants who selected each response option as a portion of all participants in that subgroup

### Brand comparison: Fitbit vs Garmin

Analyses were performed to determine whether users’ experiences and perceptions relating to activity tracker varied by brand. Only Fitbit and Garmin were included in these comparisons, since other brands had insufficient sample sizes. Results suggested that the perceived usefulness and accuracy of activity data did not vary between brands (Additional file 3: Table S2). However, some aspects of ease of use did vary; specifically, Fitbit users rated the stair climbing, heart rate and dietary intake features as being significantly easier to use than Garmin users did (*p* = 0.01–0.02).

There was no significant difference between the total number of complaints reported by Fitbit users (*Mdn =* 2.00) and Garmin (*Mdn* = 1.50) users (*U* = 1419.00, *z* = −1.36, *p* = .17, *r =* .11). However, Fitbit users were more likely to report complaints relating to their activity trackers’ battery life (*X* ^2^ (1) = 12.59, *p* < .01), whilst Garmin users were more likely to report problems with their activity trackers’ screens (*X* ^2^ (1) = 4.89, *p <* .05; Additional file 4: Table S3).

## Discussion

This study explored users’ experiences of wearable activity trackers, and in doing so, contributes a range of new insights to the growing body of literature regarding wearables. The study found that in general, activity trackers are used for a substantial period of time and are viewed positively by users. The majority of users perceived they had increased their physical activity as a result of using the activity tracker. Key barriers to continued use were device breakage or loss, and technical difficulties with the device/accompanying software.

On average, current users reported they had worn their device for 7 months and most were planning to continue using their device for three more years. These findings are consistent with previous work that has found some segments of users who have used, or intend to use, their device for a sustained period of time [10–12]. In contrast, former users wore their devices for only 5 months and close to one third of these users cited having learnt everything they could as their reason for stopping. Emerging evidence suggests that some users are able to quickly gain a good understanding of their activity levels, and no longer feel a need to monitor themselves. It has been suggested that this may be particularly true for users who were adequately active prior to using their device [10, 11]. One third of users stopped because their device either broke or had technical issues, highlighting that these issues are both relatively common and major barriers to continued use. The most commonly reported complaint amongst all users was low battery life. In this regard, the Garmin Vivofit range offers a major advantage, with a ≥ 1 year battery life.

The majority of participants reported that they did not share their activity data to online social networks, whilst one third shared their data within the software associated with their device. A previous qualitative analysis suggests that in the early stages of device use, users are more likely to share their data with real-life friends and family, and then evolve towards a preference to share with other users who hold similar health goals, interests or attributes both in person and through online health-related social network communities [10]. Similarly, Chang et al. [13] describe a preference to share data within a social network of people who are specifically interested in physical activity. In particular, users may seek to compare their own data against a baseline aggregated from users who share similar attributes to themselves, to allow them to gain a clear understanding of their own activity levels without the need to broadcast to social media [14]. As with previous work, the current study found that only a small number of users shared data to Facebook (3.5%), Instagram (1%) or Twitter (0.5%). This may reflect a move away from traditional social media, and towards a preference to share on platforms designed to bring together groups of people who are interested in health [15], for example the second most common place for users in the current study to share data was to Strava (3.5%), a special-interest social media platform that focusses exclusively on running and cycling.

The study has a number of strengths. Firstly, to the best of our knowledge, this is one of the first studies to explore activity trackers from the perspective of the end user and therefore provides important insights into their experiences and preferences. Given that this is a new field of research, no suitable pre-existing survey instruments were available, so a survey was purpose-designed for this study. The survey instrument was developed using a rigorous process, including seeking feedback from independent experts in the field who confirmed face validity, and undergoing pilot testing within the target demographic, including test-retest reliability evaluation (albeit with a small sample of *n* = 11). Further strengths are that the study captured the views of both current and former users, and in doing so, was able to highlight some differences in the experiences and preferences between these groups.

It is difficult to know how generalizable the results are, since it is impossible to track how widely, and to which Facebook users, the survey link was shared and the sample was self-selecting. Participants were encouraged to on-share the link with an incentive offered to do so, consisting of an additional entry to the prize draw. The research team disseminated the survey link by posting it to eight Facebook groups (including a mix of local and national groups) and in addition, 31 participants shared the Facebook link to their personal profiles. The use of Facebook as a recruitment platform may have introduced an age bias, since older adults are under-represented on this platform. An additional limitation is that the study’s explorative, cross-sectional design increases the risk of recall bias, particularly in relation to whether users have truly changed their behaviour as a result of their activity tracker, and the experiences of former activity tracker users. Finally, Fitbit was used by the vast majority of users in the study and it was therefore difficult to make comparisons across device brands and models. Future research should address these limitations by recruiting a larger sample of participants to ensure they are representative of a typical user and that each major brand of wearable is represented.

Whilst activity trackers are capable of collecting data on a wide range of activities (physical activity, diet and sleep), physical activity emerged as the most popular activity users were monitoring, and also the activity that users were most likely to report changing as a result of their tracker. Interestingly, of the variety of physical activity metrics available, step counting was the most popular. In addition, users favoured the trackers’ real-time/short term feedback over their ability for long-term monitoring. Taken together, findings suggest that activity trackers are essentially being used as pedometers. This is somewhat surprising given that they are far more expensive that conventional pedometers.

It is important to consider, however, that users in this study had used, and were intending to continue using, their activity trackers for periods of time far longer than people tend to use conventional pedometers [16]. This suggests that aspects of activity trackers offer important advantages over traditional pedometers, and it is interesting to briefly consider these. Key advantages may include that modern day activity trackers tend to be wrist-worn, which literature has identified as improving compliance compared with waist-worn devices [11]. Modern activity trackers are visually attractive and aesthetically pleasing, which may increase compliance compared with traditional pedometers. Furthermore, it is possible that while a sizeable portion of users reported that they did not use their devices to monitor sleep or diet and that they placed less emphasis on the value of the long-term monitoring compared with real-time feedback, the availability of this suite of metrics and features may increase the appeal, and therefore long-term usage, of modern activity trackers. The convenience offered by activity trackers in terms of their ability to automatically track and store health data may contribute further to their appeal, particularly in comparison with non-smart methods of tracking activity (e.g. non-smart pedometers and diaries).

The majority of respondents in this study used Fitbit and Garmin devices, preventing thorough comparison of users’ experiences across a range of manufacturers. Findings from the current study suggest that overall satisfaction issues with Fitbit and Garmin devices are reasonably similar, although Fitbit devices may be slightly easier to use whilst Garmin devices have the advantage of long battery life. Overall, satisfaction levels were high, suggesting that numerous manufacturers are providing appealing activity tracker offerings.

## Conclusions

This study explored users’ perceptions and experiences with wearable activity trackers, finding that activity trackers are generally used for a substantial period of time and are viewed positively by users. Participants predominantly use their trackers to monitor and intervene on physical activity rather than other daily activities (e.g. sleep and diet) and were slightly more likely to value the trackers’ real-time feedback more than long term monitoring capabilities. The majority of users perceived they had increased their physical activity as a result of using the activity tracker. Key barriers to continued use were device breakage or loss and technical difficulties with the device/accompanying software. Taken together, findings support activity trackers as appealing and useful tools for intervening on physical activity.

## Additional files

Additional file 1: Supplementary Material 4 survey. Survey instrument. Survey instrument that participants completed. (PDF 274 kb)

Additional file 2: Table S1. Participants’ primary motivation for wearing an activity tracker. Table displaying the number and percentage of participants who selected one of seven motivation domains as their primary motivation for wearing an activity tracker. (DOCX 13 kb)

Additional file 3: Table S2. Fitbit and Garmin users’ evaluation of activity tracker features. Table displaying users’ ratings of the ease of use, usefulness, and accuracy of activity tracker features, segregated by brand (Fitbit and Garmin). (DOCX 15 kb)

Additional file 4: Table S3. Complaints reported by Fitbit and Garmin users. Table displaying the number and percentage of participants who reported to experience a range of complaints related to their activity tracker. (DOCX 13 kb)

## Abbreviations

app.: application
Mdn: median
n: number of participants in subgroup
N: number of participants in whole sample
r: correlation coefficient
SD: standard deviation
TAFE: Technical and Further Education
U (Mann-Whitney U test): Unbiased
US: United States
z: z-test score
