# Supplementary material for: Users’ experiences of wearable activity trackers: a cross-sectional study
Source: BMC Public Health. 2017 Nov 15;17:880. doi: 10.1186/s12889-017-4888-1 (PMC5688726; doi:10.1186/s12889-017-4888-1)
Supplement: Supplementary file 1 — Survey instrument. Survey instrument that participants completed. (PDF 274 kb) [file 12889_2017_4888_MOESM1_ESM.pdf]

Question	Response option/s
**ALL USERS**	
1. **Before you got your activity tracker, in a typical week, on how many days did you get 30 minutes of physical activity?**	Open field, numerical
2. **In the last week, how many days did you get 30 minutes of physical activity?**	Open field, numerical
3. **What is the highest level of education that you have completed?**	• Primary School<br>• High School<br>• [TAFE/certificate/diploma/apprenticeship]<br>• Undergraduate bachelor degree<br>• Post graduate degree
4. **Sex**	• Male<br>• Female
5. **How old are you?**	Open field, numerical
6. **What is your relationship status?**	• In a relationship<br>• Single<br>• Prefer not to say
7. **[If answered 'In a relationship' to Q6] Does your spouse or partner have an activity tracker?**	• Yes<br>• No<br>• Planning on getting one<br>• N/A
8. **What is your postcode?**	Open field, numerical
9. **Are you currently using a wearable?**	• Yes, I'm currently using a wearable<br>• No, I previously used a wearable
10. **How did you get your wearable?**	• I bought it for myself<br>• Gift from friend/family<br>• Provided by health insurer<br>• Provided by workplace
**FORMER USERS ONLY**	
11. **What is the reason you stopped using your wearable? Tick all that apply**	• It broke<br>• Got lost<br>• It was difficult to understand<br>• Technical difficulties<br>• It wasn't helping with my goals<br>• I didn't like it<br>• I found it intrusive<br>• I was experiencing negative psychological impacts<br>• I learnt everything I could from it<br>• Other (please specify)
12. **Which wearable did you use?**	Open field, free text

Question	Response option/s
13. **Did you have any complaints with your wearable? Tick all that apply.**	• None<br>• Technical issues<br>• It falls off<br>• It doesn't fit<br>• It is uncomfortable<br>• Lost it<br>• Low battery life<br>• General wear and tear<br>• It often does not match my outfit<br>• Problems with the screen<br>• Problems uploading the data to supporting software<br>• Problems interpreting the data<br>• Problems with navigation of supporting website and technology<br>• Inaccurate at recording data<br>• Other (please specify)
14. **Overall, I had a positive experience using my wearable.**	• Disagree strongly<br>• Somewhat disagree<br>• Neutral<br>• Somewhat agree<br>• Agree strongly
15. **How long (months) did you use your wearable?**	• Dropdown list ranging from <1 to >36 months
16. **What was your main motivation for using your wearable?**	• To improve fitness<br>• To improve health<br>• To improve appearance<br>• To monitor activities<br>• To share my activity<br>• To compete with family/ friends<br>• To keep up with new technology<br>• Other (please specify)
17. **Which graph best matches how your activity levels changed after getting your wearable?**<br>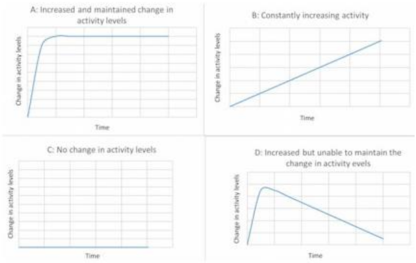 	• A<br>• B<br>• C<br>• D<br>• Other (please specify)
18. **How much do you agree/disagree with the following statements? Since using my wearable I now...**<br>• Eat healthier<br>• Incorporate more activity in my day<br>• Sleep more	• Disagree strongly<br>• Somewhat disagree<br>• Neutral<br>• Somewhat agree<br>• Agree strongly

Question	Response option/s
19. **How much do you agree/disagree with the following statements? I found the ....** monitoring on my wearable useful.<br>• Real time<br>• Long term	• Disagree strongly<br>• Somewhat disagree<br>• Neutral<br>• Somewhat agree<br>• Agree strongly
20. **How much do you agree/disagree with the following statements? When I used my wearable it made me feel....**<br>• Guilty<br>• Empowered<br>• Motivated<br>• Accountable<br>• Self-conscious<br>• Anxious	• Disagree strongly<br>• Somewhat disagree<br>• Neutral<br>• Somewhat agree<br>• Agree strongly
21. **How much do you agree/disagree with the following statements. When I didn't use/forgot/couldnt use my wearable it made me feel....**<br>• Guilty<br>• Frustrated<br>• Anxious<br>• Liberated	• Disagree strongly<br>• Somewhat disagree<br>• Neutral<br>• Somewhat agree<br>• Agree strongly
**CURRENT USERS ONLY**	
22. **How much do you agree/disagree with the following statements? I find the following features on my wearable easy to use......**<br>• Active minutes<br>• Steps<br>• Stairs<br>• Sleep<br>• Heart rate<br>• Energy burned<br>• Energy consumed (food)<br>• Connection to other devices e.g. tablet or mobile phone<br>• Sharing the data e.g. on social media	• Disagree strongly<br>• Somewhat disagree<br>• Neutral<br>• Somewhat agree<br>• Agree strongly

Question	Response option/s
23. **How much do you agree/disagree with the following statements? I find the following features on my wearable useful.....**<br>• Active minutes<br>• Steps<br>• Stairs<br>• Sleep<br>• Heart rate<br>• Energy burned<br>• Energy consumed (food)	• Disagree strongly<br>• Somewhat disagree<br>• Neutral<br>• Somewhat agree<br>• Agree strongly<br>• N/A
24. **How much do you agree/disagree with the following statements? I find the following features on my wearable accurate....**<br>• Active minutes<br>• Steps<br>• Stairs<br>• Sleep<br>• Heart rate<br>• Energy consumed (food)	• Disagree strongly<br>• Somewhat disagree<br>• Neutral<br>• Somewhat agree<br>• Agree strongly<br>• N/A
25. **Following a full charge, how long does your wearable's battery last?**	Dropdown menus: Hours/days/weeks/months
26. **Do you have any complaints with your wearable? Tick all that apply.**	• None<br>• Technical issues<br>• It falls off<br>• It doesn't fit<br>• It is uncomfortable<br>• Lost it<br>• Low battery life<br>• General wear and tear<br>• It often does not match my outfit<br>• Problems with the screen<br>• Problems uploading the data to supporting software<br>• Problems interpreting the data<br>• Problems with navigation of supporting website and technology<br>• Inaccurate at recording data<br>• Other (please specify)
27. **How long (months) are you planning to continue using your wearable?**	• Dropdown list ranging from <1 to >36 months
28. **How long (months) have you been using your wearable?**	• Dropdown list ranging from <1 to >36 months

Question	Response option/s
29. **For which of the following activities do you remove your wearable? Tick all that apply**	• Work<br>• Special occasions<br>• Sport<br>• Bathing/ swimming<br>• Charging<br>• Sleep<br>• None<br>• Other (please specify)
30. **Overall, I have had a positive experience using my wearable.**	• Disagree strongly<br>• Somewhat disagree<br>• Neutral<br>• Somewhat agree<br>• Agree strongly
31. **What is your main motivation for using your wearable?**	• To improve fitness<br>• To improve health<br>• To improve appearance<br>• To monitor activities<br>• To share my activity<br>• To compete with family/ friends<br>• To keep up with new technology<br>• Other (please specify)
32. **Do you have a daily goal?**	• Yes<br>• No
33. **Please specify your daily goal:**<br>• Active minutes<br>• Steps<br>• Sleep<br>• Energy burned<br>• Energy consumed (food)<br>• Other	Open field, free text
34. **Which graph best matches how your activity levels have changed since getting your wearable?**<br>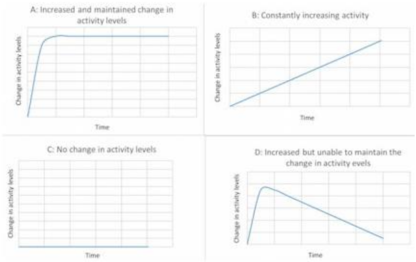 	• A<br>• B<br>• C<br>• D
35. **How much do you agree/disagree with the following statements? Since I started using my wearable I now....**<br>• Eat healthier<br>• Incorporate more activity in my day<br>• Sleep more	• Disagree strongly<br>• Somewhat disagree<br>• Neutral<br>• Somewhat agree<br>• Agree strongly

Question	Response option/s
36. **Which device(s) do you use with your wearable? Tick all that apply.**	• Smartphone<br>• Tablet<br>• Computer<br>• Laptop<br>• None
37. **What is the main device you use to track your activity?**	• Smartphone<br>• Tablet<br>• Computer<br>• Laptop<br>• None
38. **Where do you share your activity data? Tick all that apply.**	• Facebook<br>• Twitter<br>• Instagram<br>• With the wearable's software<br>• I don't share my activity data<br>• Other (please specify)
39. **Why do you share your activity on social networks? Tick all that apply.**	• To share progress<br>• To compete with friends<br>• To get encouragement from others<br>• To motivate others<br>• I don't share data<br>• Other (please specify)
40. **How many times a day do you check your progress?**	• Open field, numerical
41. How much do you agree/disagree with the **following statements? I find the .... monitoring on my wearable useful.**<br>• Real time<br>• Long-term	• Disagree strongly<br>• Somewhat disagree<br>• Neutral<br>• Somewhat agree<br>• Agree strongly
42. **How much do you agree/disagree with the following statements? When I'm using my wearable it makes me feel....**<br>• Guilty<br>• Empowered<br>• Motivated<br>• Accountable<br>• Self-conscious<br>• Anxious	• Disagree strongly<br>• Somewhat disagree<br>• Neutral<br>• Somewhat agree<br>• Agree strongly

Question	Response option/s
43. **How much do you agree/disagree with the following statements? When I'm not using/forget/can't use my wearable it makes me feel....**<br>• Guilty<br>• Frustrated<br>• Anxious<br>• Liberated	• Disagree strongly<br>• Somewhat disagree<br>• Neutral<br>• Somewhat agree<br>• Agree strongly
44. **Have you consciously cheated to record your activity on your wearable?**	• Yes (if so, how?)<br>• No
45. **Here are a number of personality traits that may or may not apply to you. Please indicate the extent to which you agree/disagree with each pair. You should rate the extent to which the pair of traits applies to you, even if one characteristic applies more strongly than the other.**<br>• Extraverted, enthusiastic<br>• Critical, quarrelsome<br>• Dependable, self-disciplined<br>• Anxious, easily upset<br>• Open to new experiences, complex<br>• Reserved, quiet<br>• Sympathetic, warm<br>• Disorganised, careless<br>• Calm, emotionally stable<br>• Conventional, uncreative	• Disagree strongly<br>• Disagree moderately<br>• Disagree a little<br>• Neutral<br>• Agree a little<br>• Agree moderately<br>• Agree strongly

END
