# Supplementary material for: Users’ experiences of wearable activity trackers: a cross-sectional study
Source: BMC Public Health. 2017 Nov 15;17:880. doi: 10.1186/s12889-017-4888-1 (PMC5688726; doi:10.1186/s12889-017-4888-1)
Supplement: Supplementary file 2 — Participants’ primary motivation for wearing an activity tracker. Table displaying the number and percentage of participants who selected one of seven motivation domains as their primary motivation for wearing an activity tracker. (DOCX 13 kb) [file 12889_2017_4888_MOESM2_ESM.docx]

**Supplementary Table 1. Participants’ primary motivation for wearing an activity tracker.**

| **Motivation** | **Former**  **(n = 37)**  ***n* (%)** | **Current**  **(n = 200)**  ***n* (%)** | **Total**  **(n = 237)**  ***n* (%)** | **Significance of between group differences**  ***p*** |
| --- | --- | --- | --- | --- |
| Monitor activities | 11 (29.7%) | 74 (37.0%) | 85 (35.9%) | .35 |
| Improve fitness | 14 (37.8%) | 51 (25.5%) | 65 (27.4%) | .13 |
| Improve health | 3 (8.1%) | 40 (20.0%) | 43 (18.1%) | .08 |
| Improve appearance | 1 (2.7%) | 1 (0.5%) | 2 (0.8%) | .18 |
| Compete with family & friends | 1 (2.7%) | 6 (3.0%) | 7 (2.9%) | .91 |
| To keep up with technology | - | 4 (2.0%) | 4 (1.7%) | .38 |
| Other | 5 (13.5%) | 10 (5.0%) | 15 (6.3%) | .06 |

Note: Participants were allowed to select multiple responses, and percentages reflect the number of participants who selected each response option as a portion of all participants in that subgroup.
