# Supplementary material for: Users’ experiences of wearable activity trackers: a cross-sectional study
Source: BMC Public Health. 2017 Nov 15;17:880. doi: 10.1186/s12889-017-4888-1 (PMC5688726; doi:10.1186/s12889-017-4888-1)
Supplement: Supplementary file 3 — Fitbit and Garmin users’ evaluation of activity tracker features. Table displaying users’ ratings of the ease of use, usefulness, and accuracy of activity tracker features, segregated by brand (Fitbit and Garmin). (DOCX 15 kb) [file 12889_2017_4888_MOESM3_ESM.docx]

**Supplementary Table 2. Fitbit and Garmin users’ evaluation of activity tracker features.**

| **Feature**  **(ease of use)** | **Fitbit *Average**** | **Garmin *Average**** | ***Statistics*** |
| --- | --- | --- | --- |
| Stair climbing | *Mdn* = 4.00 | *Mdn* = 3.00 | *U* = 1072.00, *z* = -2.42, *p* = **.02***, *r* = .20 |
| Heart rate | *Mdn* = 5.00 | *Mdn* = 4.00 | *U* = 1091.00, *z* = -2.54, *p* = .**01***, *r* = .22 |
| Energy consumed | *M* = 3.12 *SD* = 1.21 | *M* = 2.55 *SD* = 1.12 | *t*(132) = 2.29, *p* = .**02***, *g* = .48 |
| Energy burned | *Mdn* = 4.00 | *Mdn* = 4.00 | *U* = 2236.50, *z* = -.36, *p* = .72, *r* = .03 |
| Active minutes | *Mdn* = 4.00 | *Mdn* = 4.00 | *U* = 1761.50, *z* = -1.69, *p* = .09, *r* = .13 |
| Step counts | *Mdn* = 5.00 | *Mdn* = 5.00 | *U* = 2265.00, *z* = -.42, *p* = .675, *r* = .03 |
| Sleep | *Mdn* = 4.00 | *Mdn* = 4.00 | *U* = 2192.50, *z* = -.25, *p* = 0.81, *r* = .02 |
| Connecting to other devices | *Mdn* = 5.00 | *Mdn* = 5.00 | *U* = 2184.50, *z* = -.67, *p* = .50, *r* = .05 |
| Sharing data | *M* = 3.681 *SD* = 1.059 | *M* = 3.548 *SD* = 1.287 | *t*(123) = .57, *p* = .57, *g* = .12 |
| **Feature (usefulness)** |  |  |  |
| Stair climbing | *Mdn* = 4.00 | *Mdn* = 4.00 | *U* = 1453.00, *z* = -.32, *p* = .75, *r* = .03 |
| Heart rate | *Mdn* = 5.00 | *Mdn* = 4.00 | *U* = 1412.00, *z* = -.30, *p* = .19, *r* = .11 |
| Energy consumed | *M* = 3.07  *SD* = 1.26 | *M* = 3.19  *SD* = 1.33 | *t*(131) = -0.42, *p* = .67, *g* = .09 |
| Energy burned | *Mdn* = 4.0 | *Mdn* = 4.50 | *U* = 1904.50*, z* = -1.52, *p* = .13, *r* = .12 |
| Active minutes | *Mdn* = 5.00 | *Mdn* = 4.00 | *U* = 1632.00, *z* = -1.71, *p* = .09, *r* =.14 |
| Step counts | *Mdn* = 5.00 | *Mdn* = 5.00 | *U* = 2312.00, *z* = -.14, *p* = .89, *r* = .01 |
| Sleep | *Mdn* = 4.00 | *Mdn* = 4.00 | *U* = 2018.00, *z* = -.66, *p* = .51, *r* = .05 |
| **Feature (accuracy)** |  |  |  |
| Stair climbing | *M* = 3.31  *SD* = 1.22 | *M* = 3.33  *SD* = 1.24 | *t*(141) = -0.08, *p* = .94, *g* = .68 |
| Heart rate | *Mdn* = 4.00 | *Mdn* = 4.00 | *U* = 1319.00, *z* = -1.09, *p* = .28, *r* = .09 |
| Energy consumed | *M* = 3.22  *SD* = 1.02 | *M* = 2.96  *SD* = 1.19 | *t*(119) = 1.10, *p* = .27, *g* = .25 |
| Energy burned | *M* = 3.47  *SD* = 1.02 | *M* = 3.82  *SD* = 0.94 | *t*(157) = -1.81, *p* = .07, *g* = .35 |
| Active minutes | *Mdn* = 4.00 | *Mdn* = 4.00 | *U* = 1776.00, *z* = -0.45, *p* = .66, *r* = .04 |
| Step counts | *Mdn* = 4.00 | *Mdn* = 4.00 | *U* = 2131.50, *z* = -0.89, *p* = .37, *r* = .07 |
| Sleep | *M* = 3.46  *SD* = 1.16 | *M* = 3.63  *SD* = 1.09 | *t*(154) = -0.757, *p* = .45, *g* = .15 |

Note *Potential scores range from 1 – 5, corresponding with response options of 1 = disagree strongly, 2 = somewhat disagree, 3 = neutral, 4 = somewhat agree, 5 = agree strongly.
