# Supplementary material for: Users’ experiences of wearable activity trackers: a cross-sectional study
Source: BMC Public Health. 2017 Nov 15;17:880. doi: 10.1186/s12889-017-4888-1 (PMC5688726; doi:10.1186/s12889-017-4888-1)
Supplement: Supplementary file 4 — Complaints reported by Fitbit and Garmin users. Table displaying the number and percentage of participants who reported to experience a range of complaints related to their activity tracker. (DOCX 13 kb) [file 12889_2017_4888_MOESM4_ESM.docx]

**Supplementary Table 3. Complaints reported by Fitbit and Garmin users.**

| **Complaints** | **Fitbit *n* (%)** | **Garmin *n* (%)** | **Significance of between group differences**  ***p*** |
| --- | --- | --- | --- |
| None | 44 (27.5%) | 11 (28.2%) | .93 |
| Low battery life | 41(25.6%) | - | **<.01*** |
| Technical issues | 20 (12.5%) | 6 (15.4%) | .63 |
| Falls off | 12 (7.5%) | 4 (10.3%) | .52 |
| Uncomfortable | 15 (9.4%) | 2 (5.1%) | .53 |
| Lost | 5 (3.1%) | - | .59 |
| General wear and tear | 26 (16.3%) | 3 (7.7%) | .17 |
| Does not match outfit | 30 (18.8%) | 8 (20.5%) | .80 |
| Problems with the screen | 4 (2.5%) | 4 (10.3%) | **<.05* (.049)** |
| Problems uploading data | 27 (16.9%) | 8 (20.5%) | .59 |
| Problems interpreting data | 6 (3.8%) | 1 (2.6%) | .72 |
| Problems navigating the supporting website/technology | 13 (8.1%) | 2 (5.1%) | .74 |
| Inaccurate at recording data | 29 (18.1%) | 6 (15.4%) | .69 |
| Skin irritation | 6 (3.8%) | - | .60 |
| Cleaning | 3 (1.9%) | - | 1.0 |
| Waterproof | 2 (1.3%) | 1 (2.6%) |  |
| Other | 7 (4.4%) | 1 (2.6%) | .61 |
